# Distribution of the parasitic isopod *Tachaea chinensis* in China

**DOI:** 10.1038/s41598-019-56402-1

**Published:** 2019-12-27

**Authors:** Weibin Xu, Zhibin Han, Yuenan Xing, Xin Li, Yingying Zhao, Qijun Chen, Yingdong Li

**Affiliations:** College of Animal Science and Veterinary Medicine, Shenyang Agricultural University, Dongling Road 120, Shenyang, 110866 China

**Keywords:** Biogeography, Marine biology

## Abstract

*Tachaea chinensis* Thielemann, 1910 (Isopoda: Corallanidae) is a branchial ectoparasite that attaches onto shrimps and prawns. However, the distribution of *T. chinensis* in China, especially its epidemiology, remains unclear. We determined the prevalence of *T. chinensis* on the ridgetail white prawn (*Exopalaemon carinicauda* Holthuis, 1950) in Jiangsu Province. Fifty ponds in 10 shrimp farms were assessed. Isopod species were identified by morphological features and mitochondrial 16S rRNA gene analysis. A literature review was performed to determine the geographical distribution of *T. chinensis* in China. Published data revealed that *T. chinensis* was geographically distributed throughout five provinces in China, including Liaoning, Tianjin, Henan, Hubei, and Guangxi. A total of 998 *T. chinensis* were collected from 50 ridgetail white prawn ponds in Yancheng City and Rudong County. *Tachaea chinensis* prevalence ranged from 0.98% to 4.42% in Yancheng City and 0.62% to 0.92% in Rudong County. This is the first study to investigate the geographical distribution of *T. chinensis* in China and determine the prevalence of *T. chinensis* on the ridgetail white prawn in Jiangsu Province. Overall, we provide available data that fill gaps in the epidemiology of *T. chinensis*.

## Introduction

Several economically important crustaceans, such as shrimps, lobsters, and crabs, are challenged with complex and numerous diseases, including those caused by viruses, bacteria, fungi, and parasites^1^. The epidemiology of crustacean diseases has been investigated in numerous studies, which have mainly focused on bacterial and viral diseases, such as white spot syndrome^2^, hypodermal and hematopoietic necrosis^3^, bacterial septicaemia, and several rickettsia-like diseases^4^. Disease-causing parasites, such as Microsporidia members^5^ and dinoflagellates^6^, some of which have caused significant economic losses to the aquaculture industry in many countries, have also been studied. However, comparatively less research has been conducted on parasitic isopods in shrimps than other prevalent parasites.

Parasitic isopods commonly infect a variety of organisms in almost all habitats^7^. Generally, the families Bopyroidea and Cryptoniscoidea are considered to represent the vast majority of isopods that parasitise crustaceans, and account for ~7.7% of all isopods^8,9^. The family Cymothoidae is one of the largest groups of parasitic isopods, containing more than 380 species, all of which are obligate parasites on a diverse array of marine, brackish, and freshwater fishes^10–12^. *Tachaea* spp. are classified within the family Corallanidae Hansen, 1890 and the superfamily Cymothooidea Wägele, 1989. This superfamily forms a clade of families that show a gradient from commensalism and micropredation in the families Corallanidae, Aegidae, and Tridentellidae, to obligate parasitism in Cymothoidae^10^. Corallanidae are also generally considered to be fish parasites^13,14^, but *Tachaea* spp. are thought to be ectoparasites of shrimps, with a few species being commensals of sponges. A new species, *Tachaea caridophaga*, has been reported to occur on caridean shrimps in Australia^13^. *Tachaea spongillicola* has been found to infect *Macrobrachium* spp., a common shrimp in a freshwater river system in south-eastern India. In addition, *T. spongillicola* has been collected as a commensal of the freshwater sponges *Spongilla carteri* and *S. lacustris*^15^.

*Tachaea chinensis* Thielemann (Isopoda: Corallanidae), first reported in 1910, is a branchial ectoparasite on shrimp species. To the best of our knowledge, only a few studies have been conducted on *T. chinensis* in China, and it only infects the cultured white shrimp *Litopenaeus vannamei* and *Macrobrachium* spp.^16,17^. During 2016–2017, abundant *T. chinensis* individuals were found parasitising cultured *Palaemonetes sinensis* in the rice fields of Panjin, Liaoning Province, which led to the slow growth and eventual death of shrimps^18^. Moreover, *T. chinensis* was recently found to parasitise *Exopalaemon carinicauda*, one of the economically important cultured shrimp species in China^19^. In the present study, the geographical distribution of *T. chinensis* in China was determined. In addition, the prevalence of *T. chinensis* on the ridgetail white prawn in Jiangsu Province was investigated for the first time.

## Results

*Tachaea chinensis* has a long oval-shaped body and is approximately 2.5 times longer than the width. The cephalon is small and slightly median triangular. The compound eyes are oval with distinct margins. The antennula is short, consists of seven articles (2 handle articles and 5 whip articles), reaching the posterior margin of the cephalon. The antenna is long, consists of 19 articles (3 handle articles and 16 whip articles), extending to the middle of pereonite 3.

Pereonite 1 is slightly narrower than the head and pereonite 4 is the widest, and the width of the following tergites gradually decreases towards the seventh segment. The first three pairs of pereopods move forward and the last four pairs face backward. Each pereopod consists of six unequal segments. The last segment is sharply hooked. Pleopods are paddle-shaped, divided into an endopodite and exopodite. Uropodium and telson are flat and ectatic, collectively called the tail fan.

**Figure 1:**
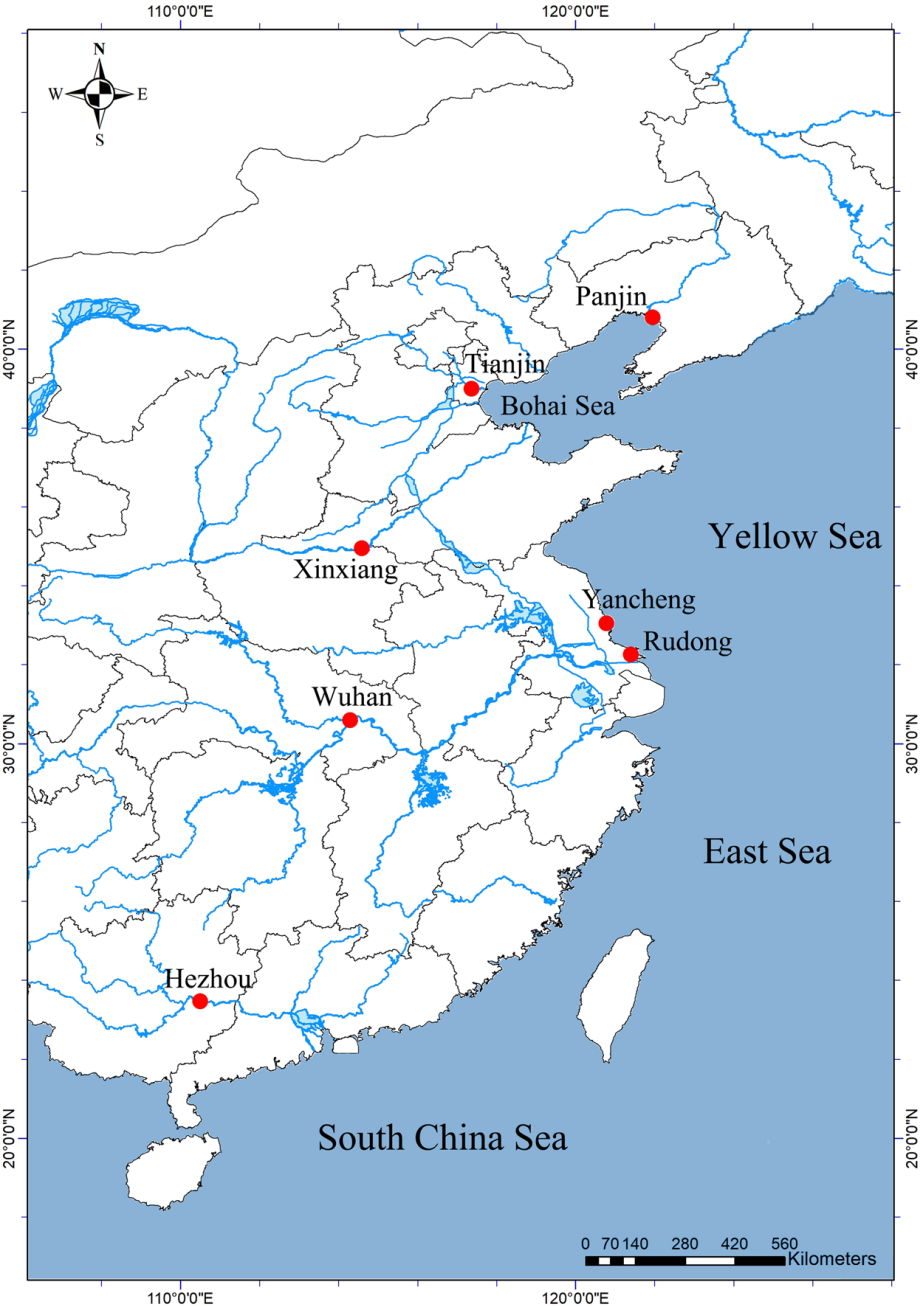
Geographical distribution of *Tachaea chinensis* in China.

The geographical distribution of *T. chinensis* in China is shown in Fig. 1. To the best of our knowledge, we included all current available data in our analysis of *T. chinensis* geographical distribution in China. The results of the literature review indicate that *T. chinensis* occurs in four regions in China, namely Tianjin City, Hebei Province^16^; Xinxiang City, Henan Province^17^; Panjin City, Liaoning Province^18^; and Wuhan City, Hubei Province^20^. An additional article described *Tachaea* sp. in Guangxi Province, but the species was unknown^21^. Furthermore, another study reviewed the distribution of *T. chinensis* in Japan^22^, but no report on the occurrence of this species in other regions of the world was found. Our research confirmed the occurrence of *T. chinensis* in Yancheng City and Rudong County, Jiangsu Province, and we herein present the first report of *T. chinensis* in this province.

In total, 998 *T. chinensis* individuals were collected from cultured ridgetail white prawn ponds in Jiangsu Province, China (Table 1). Isopod prevalence differed among two regions, with Yancheng City (0.98–4.42%) presenting a higher total prevalence than Rudong County (0.62–0.92%). The highest prevalence (4.42%) was found in farm 4 in Yancheng City and the lowest (0.62%) in farm 8 in Rudong County. In addition, the prevalence of isopod infection was 11.4% on *P. sinensis* in a rice field in Panjin City, Liaoning Province.

**Table 1:** Prevalence of *T. chinensis* in Jiangsu Province (Yancheng City and Rudong County) in November 2018.

Region	Sampling farm	Number of sampling ponds	Number of infected ponds	Number of sampling shrimps	Number of infected shrimps	Number of isopods	Prevalence of isopods (%)
Yancheng	1	5	5	5000	109	130	2.18
	2	5	5	5000	137	155	2.74
	3	5	5	5000	49	62	0.98
	4	5	5	5000	221	265	4.42
	5	5	5	5000	56	65	1.12
	6	5	5	5000	74	85	1.48
	7	5	5	5000	91	105	1.82
Rudong	8	5	5	5000	31	40	0.62
	9	5	3	5000	32	36	0.64
	10	5	3	5000	46	55	0.92
Panjin	—	—	—	656	75	82	11.4

**Figure 2:**
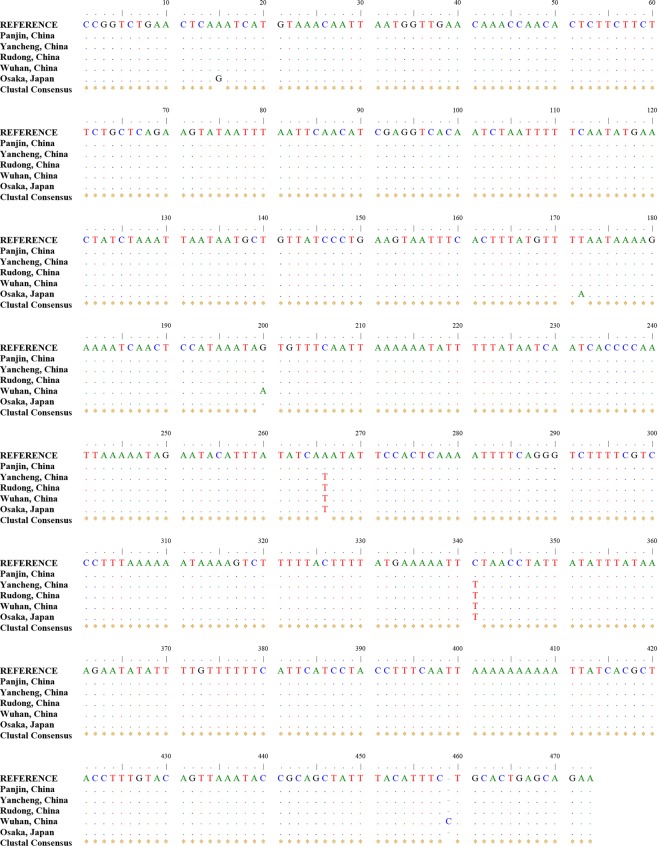
Partial mitochondrial 16S rRNA gene fragment sequences of five different populations of *Tachaea chinensis* (473 bp). The dots indicate sites identical to those in the “REFERENCE” nucleotide sequence. The asterisks denote Clustal consensus sites.

The partial mitochondrial 16S rRNA gene fragment was successfully amplified from the total DNA, and ~730 bp of DNA sequences was obtained. A 473-bp sequence was then obtained by manual calibration for the subsequent analysis. The sequences of five of the tested populations (including those in Japan and Wuhan) were aligned with the reference sequence using Clustal Omega (Fig. 2). The results confirmed that the isopods that were collected from Panjin, Yancheng, and Rudong were *T. chinensis*.

## Discussion

In this study, 998 *T. chinensis* individuals were collected from cultured ridgetail white prawn ponds in Jiangsu Province, China. During the sampling process, a hauling net was used, and the mesh was too big, making it difficult to catch free-living *T. chinensis* individuals; therefore, *T. chinensis* individuals that we collected were considered to be attached on the shrimps. Furthermore, as an ectoparasite, *T. chinensis* easily escapes from the host surface due to external stimuli. Therefore, the actual prevalence of *T. chinensis* must be considerably higher than that recorded in our study. The prevalence of parasitic isopods is considered to be driven by complex factors, including the abundance of their specific hosts and multiple ecological factors, such as season, temperature, and salinity^23,24^. In the present study, the prevalence differed among two regions, with Yancheng City (0.98–4.42%) presenting a higher total prevalence than that in Rudong County (0.62–0.92%). The highest prevalence (4.42%) was found in farm 4 in Yancheng City and the lowest (0.62%) in farm 8 in Rudong County. Although we did not analyse the correlation between prevalence and ecological factors, in shrimp farming, quicklime is often used as a disinfectant to clear ponds. The concentration of quicklime and the frequency of disinfection seem to be important factors affecting the occurrence of *T. chinensis*.

*Tachaea chinensis* showed a strong adaptability to different environments, especially variations in salinity, a factor that is considered important in defining the prevalence of parasites in different regions. In the present study, *T. chinensis* was found to occur in different environmental conditions, including freshwater and saline water. The salinity in Yancheng and Rudong areas was 25‰ and 20‰, respectively. The salinity tolerance of other isopod species, such as *Sphaeroma serratum*, which usually inhabit coastal marine or brackish waters, has been studied, and the results showed that *S. serratum* is euryhaline, with lower and upper limits of ~2‰ and 74‰, respectively^25^. *Tachaea chinensis* not only has a strong salinity tolerance in different regions, but also survives in freshwater environments such as rivers and rice fields^26,27^. Although the salinity adaptation mechanisms of *T. chinensis* are unclear, these abilities are important for the adaptation of the species to different environmental conditions.

The distribution pattern of parasitic isopods is considered to be related to their host specificity. The distribution of parasitic isopods in carangid fishes have been investigated along the southeast coast of India, and the results revealed that isopods with a broad host diversity have a potentially wider distribution than species with a narrow host diversity^28^. A study on *Cymothoa indica* (Isopoda: Cymothoidae) revealed that it parasitises a variety of fish hosts and that it also exhibits a wide geographic and host distribution^29^. In Japan, *T. chinensis* has been found in more than 30 localities in 15 prefectures and infected seven nominal shrimp and prawn species, and the results also showed that *T. chinensis* seems to have a diverse choice of hosts in different regions^22^. Furthermore, a general trend has been observed, whereby host specificity decreases with increasing latitude^10^. For example, *Anilocra physodes* (Isopoda: Cymothoidae), which inhabits high-latitude regions, infects a wider array of host species than tropical species; tropical *Anilocra* spp. typically primarily parasitises fish of a single family, or possibly a single fish species^10^. In this study, we revealed that *T. chinensis* has a low host specificity in China, including *P. sinensis*, *E. carinicauda*, *M. nipponense*, and *L. vannamei*. This may be related to the generally lower diversity of parasitic isopods in temperate regions, which reduces the competition for host species and results in an increased number of host species used.

Certain host behaviours, such as migration, may also affect the distribution of parasites. Some migratory river shrimps, such as *Macrobrachium ohione*, inhabit the Mississippi River and migrate from the upriver adult habitat down to brackish-water estuaries to release hatching larvae. Some of the shrimps are parasitised by the bopyrid parasite *Probopyrus pandalicola* in estuarine areas. When adults migrate back to the freshwater habitat, some of the returning shrimps are infected with bopyrid parasites^30^. In the present study, the areas that we surveyed were mostly located in estuaries, where water is transferred between the rivers and the sea. *Tachaea chinensis* from the infested ponds probably entered the natural water system via the drainage systems from the ponds. In this process, isopods were able to invade wild shrimps and be transported to upriver habitats on the migrating shrimps.

In the present study, *T. chinensis* was found to inhabit freshwater and seawater environments in different regions. A low host specificity is an important factor affecting the geographical distribution of *T. chinensis*, and host migration can also help transfer parasites into a new environment. In addition, the prevalence of *T. chinensis* on the ridgetail white prawn *E. carinicauda* in Jiangsu Province was firstly determined, and the prevalence of *T. chinensis* in aquaculture environments is not only affected by human factors, but also some more complex environmental factors, which requires further research.

## Materials and Methods

### Literature research

We conducted a systematic review of published papers for information on the occurrence, prevalence, and geographical distribution of isopod parasites in China from 1989 to 2019.

### Sampling procedures

Sampling was conducted in July and November 2018. Fifty ponds in 10 shrimp farms were assessed in Jiangsu Province, China, and 1000 shrimps were collected in each pond by fishermen with the use of a hauling net (mesh size, 1 cm; length, 10 m; width, 3 m). Isopods were collected on the surface of shrimps, on the net, and from the bottom of the collection baskets. In addition, isopods were collected from a rice field in Panjin City, Liaoning Province, where Chinese grass shrimps (*P. sinensis*) are cultured. During rice field samplings, a large circular hand net (mesh size, 1 mm; frame radius, 14 cm; handle length, 2 m) was used, and the shrimps were captured mainly in the shallow waters rich in aquatic plants, where *P. sinensis* individuals were mainly hidden. Details on the location, host species, and environmental characteristics of the isopod collection sites are shown in Table 2.

**Table 2:** Geographical distribution of *T. chinensis* in China.

Region	Coordinates	Host species	Environment	Sampling/Reporting time	Temperature Range (°C)	Salinity (‰)	Reference
Panjin	40° 48′ 30″N,121° 58′ 18″E	*Palaemonetes sinensis*	rice field	July 2018	24–26	0	^18^
Yancheng	33° 03′ 26″N,120° 48′ 16″E	*Exopalaemon carinicauda*	pond	November 2018	22–24	25	—
Rudong	32° 16′ 56″N,121° 25′ 23″E	*Exopalaemon carinicauda*	pond	November 2018	23–26	20	—
Tianjin	39° 00′ 00″N,117° 22′ 23″E	*Litopenaeus vannamei*	pond	2007	—	—	^16^
Xinxiang	34° 57′ 52″N,114° 36′ 09″E	*Macrobrachium nipponense*	river	2010	—	0	^17^
Wuhan	30° 34′ 42″N,114° 19′ 59″E	*Macrobrachium nipponense*	river	2018	—	0	^20^
Hezhou	23° 28′ 00″N,110° 30′ 00″E	*Macrobrachium nipponense*	river	2006	—	0	^21^

In Yancheng City, Jiangsu Province, 867 isopods were collected from all 35 ponds in seven shrimp farms. Water temperature varied from 22 °C to 24 °C and the salinity ranged from 24‰ to 26‰. In Rudong County, Jiangsu Province, 131 isopods were collected from three shrimp farms, where 13 of the 15 ponds were infected. Water temperature varied from 23 °C to 26 °C and the salinity ranged from 18‰ to 20‰. In Panjin City, Liaoning Province, 656 *P. sinensis* were collected in a field, of which 75 were infected with isopod parasites. All the collected isopods in all the three regions were carefully observed for morphological features, and species were initially recognised. Moreover, a group of isopods (n = 10) was randomly selected, stored in 75% ethanol, and then transported to the aquaculture laboratory in Shenyang Agricultural University for further identification.

### Species identification by mitochondrial 16S rRNA gene analysis

The total DNA was extracted using the TIANamp Genomic DNA kit (TIANGEN Biotech, China) following the manufacturer’s protocol. Two samples per region were randomly selected for the amplification of 16S rRNA. The following PCR primers were designed: F: 5′-ACC TAA CCA ACC ACT ACT TCC ATT-3′, R: 5′-GGT TGG TAG AGG TAG TTT CTG CT-3′, which were complementary to the 16S rRNA regions of *T. chinensis* (GenBank: MK007965). The primers were synthesised by Shanghai Personal Biotechnology Co., Ltd. PCR amplification was carried out using a 25-μL reaction mixture that contained 1 μL of each primer, 100 ng of DNA in 1 μL, 9.5 μL of sterile distilled H_2_O, 12.5 μL of PrimeSTAR Max DNA Polymerase (TAKARA, Japan) using the Veriti^™^ 96-Well Thermal Cycler (Thermo Fisher Scientific, USA). Amplification consisted of 35 cycles at 94 °C for 30 s, 56 °C for 30 s, and 72 °C for 30 s. The amplification products were analysed by 1.2% agarose gel electrophoresis. The PCR products were sent to Shanghai Personal Biotechnology Co., Ltd. for sequencing. The resulting sequences were subjected to alignment using Clustal Omega and manually calibrated, followed by homology comparisons.

### Ethics statement

Our study did not involve endangered or protected species. In China, catching isopod does not require specific permits. Animal welfare and the relevant experiment were carried out in compliance with the guide for the care and use of laboratory animals. The experimental protocol was approved by the Animal Ethics Committee of Shenyang Agriculture University.

## Data Availability

Geographical distribution and prevalence data of *Tachaea chinensis* generated or analysed during this study are included in this published article (and its Supplementary Information Files). Sequencing data have deposited in Genbank repository and accession number is MK007965 (Sequencing data will be published when this article is published).
